# Chlorogenic Acid Inhibits *Rahnella aquatilis* KM25 Growth and Proteolytic Activity in Fish-Based Products

**DOI:** 10.3390/microorganisms11061367

**Published:** 2023-05-23

**Authors:** Kamila Myszka, Natalia Tomaś, Wojciech Juzwa, Łukasz Wolko

**Affiliations:** 1 Department of Biotechnology and Food Microbiology, Poznan University of Life Sciences, Wojska Polskiego 48, 60-627 Poznan, Poland; natalia.tomas@up.poznan.pl (N.T.); wojciech.juzwa@up.poznan.pl (W.J.); 2 Department of Biochemistry and Biotechnology, Poznan University of Life Sciences, Dojazd 11, 60-632 Poznan, Poland; lukasz.wolko@up.poznan.pl

**Keywords:** spoilage of fish-based products, *Rahnella aquatilis*, chlorogenic acid, proliferation of cells, proteolytic activity

## Abstract

This work verified the antiproliferative and antiproteolytic activities of chlorogenic acid against *Rahnella aquatilis* KM25, a spoilage organism of raw salmon stored at 4 °C. Chlorogenic acid limited the growth of *R. aqatilis* KM25 in vitro at a concentration of 2.0 mg/mL. The dead (46%), viable (25%), and injured (20%) cell subpopulations were identified by flow cytometry following treatment of *R. aquatilis* KM25 with the examined agent. The exposure of *R. aquatilis* KM25 to chlorogenic acid altered its morphology. Changes in cell dimensions, mostly in length parameters from 0.778 µm to 1.09 µm, were found. The length of untreated cells ranged from 0.958 µm to 1.53 µm. The RT–qPCR experiments revealed changes in the expression of genes responsible for the proliferation and proteolytic activity of cells. Chlorogenic acid caused a significant reduction in the mRNA levels of the *ftsZ*, *ftsA*, *ftsN*, *tolB,* and *M4* genes (−2.5, −1.5, −2.0, −1.5, and −1.5, respectively). In situ experiments confirmed the potential of chlorogenic acid to limit bacterial growth. A similar effect was noted in samples treated with benzoic acid, where the growth inhibition of *R. aquatilis* KM25 was 85–95%. Reduction of microbial *R. aquatilis* KM25 proliferation significantly limited total volatile base nitrogen (TVB-N) and trimethylamine (TMA-N) formation during storage, extending the shelf life of model products. The TVB-N and TMA-N parameters did not exceed the upper levels of the maximum permissible limit of acceptability. In this work, the TVB-N and TMA-N parameters were 10–25 mg/100 g and 2.5–20.5 mg/100 g, respectively; for samples with benzoic acid-supplemented marinades, the parameters TVB-N and TMA-N were 7.5–25.0 mg/100 g and 2.0–20.0 mg/100 g, respectively. Based on the results of this work, it can be concluded that chlorogenic acid can increase the safety, shelf life, and quality of fishery products.

## 1. Introduction

Microbial spoilage of seafood remains an unsolved problem in the food industry; it usually begins after the fish is harvested and intensifies during storage of the final product. Unrestricted bacterial proliferation affects seafood waste and insecurity [1]. The initial microbial load of fresh raw fish is less than 10^6^ CFU/g; at the time of rejection of the product, it usually reaches 10^7^–10^9^ CFU/g [2]. According to the Food and Agriculture Organization (FAO), postharvest losses in the seafood sector due to microbial contamination/unchecked microbial proliferation amount to 20–35% of total fish production [3]. Therefore, effective antimicrobial solutions are needed to maintain the safety and quality of seafood and reduce food waste.

*Rahnella aquatilis* is a spoilage organism of fish-based products; these cells were found to be prevalent among others on samples of vacuum-packed salmon [4] and raw river trout [5]. The wide distribution of *R. aquatilis* in the aquatic environment and its ability to survive or even grow in products at cold temperatures make *Rahnella* spp. a concern in the seafood industry [6]. *R. aquatilis* can cause enzymatic degradation of proteins or lipids, which directly contributes to adverse sensory changes in food and its premature spoilage.

The proliferation of cold-resistant *R. aquatilis* strains must be controlled to guarantee seafood safety. Several food preservation systems, such as the addition of salts and/or synthetic preservatives, can reduce the risk of food spoilage. However, these techniques are often associated with adverse changes in the organoleptic characteristics of foods. Moreover, synthetic preservatives, such as butylated hydroxytoluene (BHT) and butylated hydroxyanisole (BHA), cause life-threatening side effects; their use may cause allergic reactions in hypersensitive consumers and/or carcinogenic effects [7]. Therefore, it is extremely important to investigate alternative food antimicrobials.

Recent research on the use of chlorogenic acid in food technology underscores its usefulness and safety [8]. Unlike other polyphenols, chlorogenic acid does not confer a “phenolic”/“medicinal” taste or bad flavor to foods [8]; additionally, it shows strong antimutagenic and antiphlogistic activities [9]. The in vitro and in vivo data also indicate that chlorogenic acid can alleviate oxidative stress [10] and interrupt free radical chain reactions [11]. However, the mechanism by which chlorogenic acid exerts its antimicrobial activity is not yet fully understood, especially in systems that mimic food products.

The purpose of this study was to investigate the effect of specific concentrations of chlorogenic acid on the proliferation/proteolytic activity of *R. aquatilis* KM025 in in vitro and in situ experiments. The *R. aquatilis* KM25 strain was isolated from commercially available fresh salmon samples. In this study, the expression levels of genes involved in the bacterial proliferation/growth and proteolytic activity of *R. aquatilis* KM25 were evaluated. Total volatile base nitrogen (TVB-N) and trimethylamine (TMA-N) values were used to control the quality of the model salmon-based product. The antimicrobial activity of selected concentrations of chlorogenic acid was demonstrated by the macrodilution method, flow cytometry, and scanning electron microscope (SEM) observations.

## 2. Materials and Methods

### 2.1. Bacterial Strain

*R. aquatilis* KM25 was isolated from commercially available fresh salmon samples. Briefly, 10 g of product was homogenized (POCH, Gliwice, Poland) and serially diluted with sterile saline (POCH, Gliwice, Poland) before plating on eosin methylene blue agar (BD, Franklin Lakes, NJ, USA). The plates were incubated at 4 °C for 72 h. Blue–green colonies were then isolated and transferred to modified Luria–Bretani medium (LB medium), consisting of 0.5% (*w*/*v*) yeast extract (BD, USA), 1% (*w*/*v*) fish peptone (HiMedia, Thane, India), and 1% (*w*/*v*) NaCl (POCH, Gliwice, Poland), and cultured at 4 °C for 72 h. An API 20E system (BioMerieux, Craponne, France) and a restriction length polymorphism of 16S rRNA gene amplicons and sequencing were used for microbial identification. The strain was negative for urea, indole, lysine decarboxylase, arginine dihydrolase, and ornithine decarboxylase reactions. In the API 20E system, the citrate utilization and Voges–Proskauer tests were positive.

The Genome Mini AX Kit (A&A Biotechnology, Gdansk, Poland) was next used for total DNA extraction from cells according to the manufacturer’s recommendations. Sequences encoding the small subunit of rRNA were amplified by PCR by applying Perpetual OptiTaq PCR Master Mix (EURx, Gdansk, Poland) and a set of primers: 27F (5′AGAGTTTGATCMTGGCTCAG 3′) and 1492R (5′CGGTTACCTTGTTACGACTT 3′). The PCR product was then sequenced in a commercial laboratory (Genomed S.A., Warszawa, Poland). Sequences were aligned, and the contigs were subjected to BLAST for nucleotide similarity searches [12]. 

*R. aquatilis* KM25 was preserved in cryovials (MWE, Corsham, UK) at −80 °C. The strain was deposited in the Department of Biotechnology and Food Microbiology’s strain collection at Poznan University of Life Sciences and is freely available.

### 2.2. Culture Conditions

*R. aquatilis* KM25 was cultured in modified LB medium enriched with serial dilutions of chlorogenic acid (Sigma-Aldrich, Merck KGaA USA, St. Louis, MO, USA) ranging from 0.1 to 5.0 mg/mL in diamethyl sulfoxide (Sigma-Aldrich, Merck KGaA USA). *R. aquatilis* KM25 was incubated at 4 °C for 72 h. The pH of the culture medium was 5.0.

### 2.3. Determination of the Antimicrobial Activity of Chlorogenic Acid

A two-step protocol consisting of the broth macrodilution method [13] and the flow cytometric evaluation of the redox potential and viability was carried out to evaluate the antimicrobial activity of selected concentrations of chlorogenic acid (Sigma-Aldrich, Merck KGaA, USA). Using the broth macrodilution method, a 6 h culture of *R. aquatilis* KM25 was inoculated with a selected dilution of chlorogenic acid. The turbidity of the culture was adjusted with sterile saline to the equivalent of the McFarland 0.5 standard. The incubation process lasted 72 h at 4 °C. The concentration of chlorogenic acid resulting in growth inhibition was selected for further experiments. Sterile medium without and with chlorogenic acid addition served as controls.

The redox potential and viability of *R. aquatilis* KM25 exposed to chlorogenic acid concentrations selected by the macrodilution method were measured by a fluorescence-based approach with the BacLight^TM^ Redox Sensor^TM^ Green Viability Kit (Life Technologies, Carlsbad, CA, USA). A BD FACS Aria^TM^ III (BD, Franklin Lakes, NJ, USA) flow cytometer was used in the study. The samples were characterized via two nonfluorescent parameters, side scatter (SSC) and forward scatter (FSC), and two fluorescent parameters, green fluorescence (FITC detector) from the Redox Sensor^TM^ Green agent by applying a 530/30 bandpass filter and red fluorescence (PE-Texas detector) from the propidium iodine (PI) agent with a 616/23 bandpass filter. FACS DIVA software (Version 6.1.3, BD, Franklin Lakes, NJ, USA) was used to analyze the data.

### 2.4. Morphology Analyses

Morphology analyses of *R. aquatilis* KM25 exposed to selected concentrations of chlorogenic acid were performed by SEM (SU3500 Hitachi, Tokyo, Japan). One drop of *R. aquatilis* KM25 culture was placed on a carbon sticker, air-dried, and covered with gold. A low voltage (15 kV) was applied for the observation of the samples.

### 2.5. RNA Isolation and RT–qPCR Analyses

The comparative quantification method for evaluating the changes in the expression of genes responsible for the proliferation of cells was carried out as described in our previous study [14]. Table 1 presents the list of primers used in this work. Briefly, RNAprotect Bacteria Reagent (Qiagen, Germantown, MD, USA) was used for the stabilization of total RNA. A PureLink^TM^ RNA Mini Kit (Thermo Fisher Scientific, Waltham, MA, USA) and a PureLink^TM^ DNase Set (Thermo Fisher Scientific, USA) were used for the isolation and purification of RNA, respectively. The quality and quantity of RNA were evaluated on a Qubit Fluorometer 4 (Invitrogen, Waltham, MA, USA) using Qubit^TM^ XR RNA and Qubit^TM^ IQ RNA Assay Kits (Invitrogen, USA). One microgram of RNA was reverse-transcribed with a High Capacity RNA-to-cDNA Kit (Life Technologies, USA).

RT–qPCR analyses were carried out on a CFX96 device (Bio-Rad, Hercules, CA, USA) using GoTaq^TM^ Master Mix (Promega, Walldorf, Germany). The cycling conditions were as follows: initial denaturation at 95 °C for 2 min; 45 cycles of denaturation at 90 °C for 15 s; and annealing and extension at 60 °C for 1 min. A 16S rRNA gene served as a reference gene. The levels of each RNA were calculated following the 2^−ΔΔCt^ method [15].

### 2.6. In Situ Evaluation of Biopreservative Properties of Chlorogenic Acid

#### 2.6.1. Preparation of Salmon-Based Samples

Ten gram portions of raw salmon filets were inoculated with 2 mL of a *R. aquatilis* KM25 suspension containing 10^4^ CFU/mL. To allow cell attachment, the salmon filets were air dried at 22 °C for 15 min in a biosafety cabinet (Thermo Fischer Scientific, USA), marinated, and packaged. The selected concentration of chlorogenic acid (Sigma-Aldrich, Merck KGaA USA) was added to fish marinade comprising 95% olive oil and 5% vinegar. Marinade supplemented with the addition of benzoic acid (Sigma-Aldrich, Merck KGaA USA) at a concentration of 0.001 mg/mL served as a control. The marinades were then poured over the fish filets. The samples were next wrapped in polyvinyl chloride stretch films (Kraina Foils Packaging, Stanica, Poland) and stored in an incubator (Thermo Scientific, USA) set at 4 ± 1 °C for 5 days. 

#### 2.6.2. In Situ Antimicrobial Assay

Verification of *Rahnella* spp. growth on cold-stored marinated products was performed every 24 h. Ten grams of the sample were placed in polyethylene bags (Sigma-Aldrich, Merck KGaA, USA) filled with 90 mL of 0.1% sterile peptone water (Oxoid, Basingstoke, UK). After the homogenization step, 0.1 mL aliquots of serially diluted suspensions were spread in duplicate on tryptic soy agar (TSA) plates (Oxoid, UK). The plates were incubated at 4 °C for 72 h. The experiments were performed in triplicate. Salmon filets treated with marinades without the addition of chlorogenic acid/benzoic acid served as controls. The antibacterial activities of the examined agents were calculated according to Formula (1):

Bacterial growth inhibition = 100 − [A/B × 100](1)

where A represents counts of *R. aquatilis* KM25 in salmon filets with marinate supplemented with the examined agents; B indicates counts of *R. aquatilis* KM25 in the reference samples.

#### 2.6.3. Determination of TVB-N

The procedure described by Goulas and Kontominas [16] was performed. Ten gram portions of salmon filets were mixed with 250 mL of distilled water and homogenized. The mixture was quantitatively transferred into a 500 mL round-bottom flask. After the addition of 2 g of MgO (Sigma-Aldrich, Merck KGaA USA) and one drop of silicone to prevent foaming, distillation was carried out. An Erlenmeyer flask containing 25 mL of a 3% solution of boric acid (Sigma-Aldrich, Merck KGaA USA) and 0.04 mL of a mixture of methyl red and methylene blue (Sigma-Aldrich, Merck KGaA USA) was used as the distillate receiver. Distillation was performed until a final volume of 125 mL of distillate was obtained. The boric acid solution turned green when made alkaline by the distilled TVB-N. This was then titrated with an aqueous 0.1 N HCl solution (POCH, Poland). Complete neutralization was obtained when the color of the distillate turned pink upon the addition of a further drop of 0.1 N HCl solution. The quantity of TVB-N in mg/100 g of sample was calculated according to Formula (2):

mg TVB-N = ((*V*_1_ − *V*_2_) × *C* × 14 × 100)/10 (2)

where *V*_1_ is the titration volume for the tested sample; *V*_1_ is the titration volume of the reference sample; and *C* is the actual concentration of HCl.

#### 2.6.4. Determination of TMA-N

TMA-N levels (mg/100 g salmon filets) were determined by the method of Goulas and Kontominas [16] as described above, with the exception that 1 mL of 10% neutralized formalin was used to block the primary and secondary amines. The amount of TMA-N in mg/100 g of fish-based product was calculated from the volume (*V*) of HCl added and its concentration (*C*) as follows (Formula (3)):

mg TMA-N = *V* × *C* × 14 (3)

### 2.7. Statistical Analysis

The experiments were carried out in triplicate; the results are expressed as the mean ± standard deviation. To characterize the difference between particular results, Tukey’s parametric post hoc test in Statistica software was performed (Version 10.0, Statsoft, Inc., Tulsa, OK, USA). Significant differences were considered at *p* < 0.05.

## 3. Results and Discussion

### 3.1. Antimicrobial Activity of Chlorogenic Acid

Given the perishable nature of fish-based products, the search for satisfactory agents for shelf-life extension that ensure quality maintenance and a continuous supply of shelf-stable quality products has recently attracted much attention [17,18]. Health concerns over the consumption of synthetic preservatives have prompted searches for active ingredients in plants [19]. The proven biological activity of chlorogenic acid, including its antioxidant properties, liver and kidney protection, anticancer activity, and sugar regulation, makes this compound an ideal candidate for use in seafood preservation systems [19]. Chlorogenic acid can achieve bacteriostasis in seafood by interfering with normal cell cycle progression, thereby inhibiting the growth of microorganisms; however, the above activity was evaluated in a limited number of bacterial cultures [20,21]. Moreover, studies characterizing spoilage microorganisms of commercial fish-based products showed the predominant presence of *R. aquatilis* in samples [22]; the bacteria were responsible for protein/lipid degradation and for the process of reducing trimethylamine oxide (TMAO) to trimethylamine (TMA), which induces a change in the odor of catfish fillets [23]. Inhibition of the growth and metabolic activity of *Rahnella* spp. can help maintain product quality.

The antimicrobial activity of different dilutions of chlorogenic acid against *R. aquatilis* KM25 was assessed via optical density measurements. We used different concentrations of chlorogenic acid, ranging from 0.1 to 5.0 mg/mL. The 2.0 mg/mL solution inhibited *R. aquatilis* KM25 growth and was selected for further experiments. The antibacterial potential of chlorogenic acid was also evaluated by Wang et al. [24]. In that study, 10.24 mg/mL chlorogenic acid reduced *Escherichia coli* and *Pseudomonas aeruginosa* PA14 proliferation in vitro. Yang et al. [25] also showed the inhibitory effect of chlorogenic acid-grafted chitosan on a seafood-derived *Pseudomonas fluorescens* isolate. In this work, the MIC of chlorogenic acid-grafted chitosan against the fish spoiler *P. fluorescens* was 1.25 mg/mL. The duration of the measurement, the density of the starting culture, the use of optical density, or the number of cells to determine growth can cause differences in the selection of concentrations of antimicrobials in general [26].

**Figure 1:**
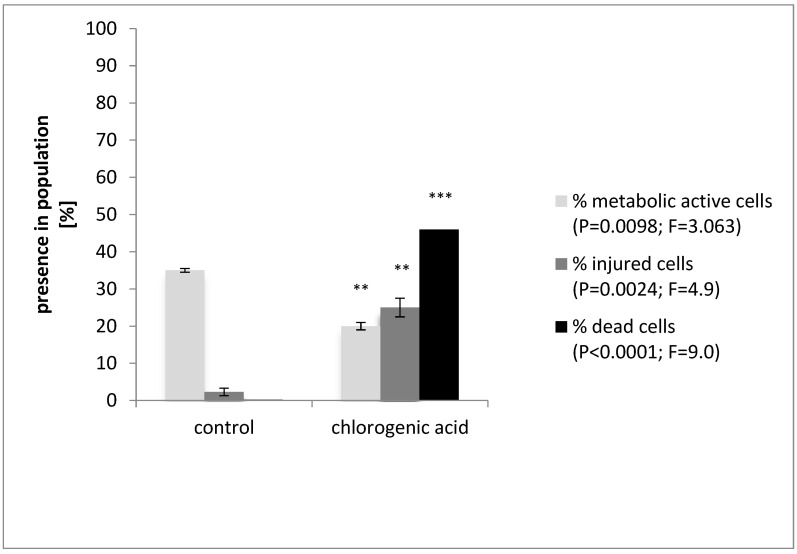
Flow cytometry analysis of *Rahnella aquatilis* KM25 cells viability following treatment with chlorogenic acid. Values are means ± SD (n = 3); ** *p* < 0.01, *** *p* < 0.001, as compared with control.

**Figure 2:**
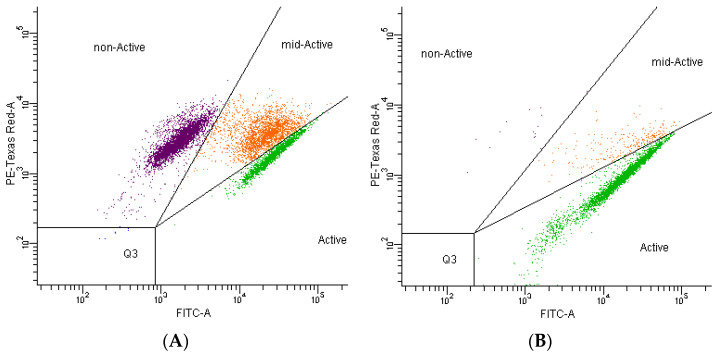
The distribution of individual cell fractions: *Rahnella aquatilis* KM25 following treatment with chlorogenic acid (**A**); control culture of *Rahnella aquatilis* KM25 (**B**).

The impact of 2.0 mg/mL chlorogenic acid on *R. aquatilis* KM25 viability and cell damage was next verified by flow cytometry. Flow cytometry is capable of generating data on the extent of heterogeneity within a population [27]. In this study, the subpopulations of dead, viable, and injured cells were identified after staining the samples with Redox-Sensor^TM^ Green and PI reagents. The flow cytometric results are shown in Figure 1 and Figure 2. The subpopulations of dead and injured *R. aquatilis* KM25 cells were dominant at 46% and 25% of the total population, respectively. In the *R. aquatilis* KM25 control sample, the percentages of dead and injured cells were 0.2% and 2.3%, respectively. Following treatment with chlorogenic acid at the selected concentration, the percentage of the subpopulation of viable *R. aquatilis* KM25 cells was 20%; for the control samples, the content of viable cells equaled 34.9% (Figure 1 and Figure 2). Similar conclusions were reached by other studies attempting to explain the antimicrobial action of phenolic-rich *Satrueja montana* and *Origanum majorana* decoctions [28]. *Staphylococcus aureus* ATCC25923 showed increased permeability to PI reagent after exposure to both decoctions, revealing a loss of membrane integrity [28]. Membrane damage can lead to compromised cellular homeostasis and, consequently, to the disturbance of normal cell function [29]. Porin proteins present in the outer membrane can create channels large enough to allow the restricted passage of molecules with a molecular mass below 600 Da, allowing their slow penetration into the periplasmic space and the cytoplasmic membrane [30]. The presence of chlorogenic acid in food can inhibit the growth of microorganisms responsible for product spoilage and pathogenic strains.

### 3.2. Effect of Chlorogenic Acid on Morphology of Cells

**Figure 3:**
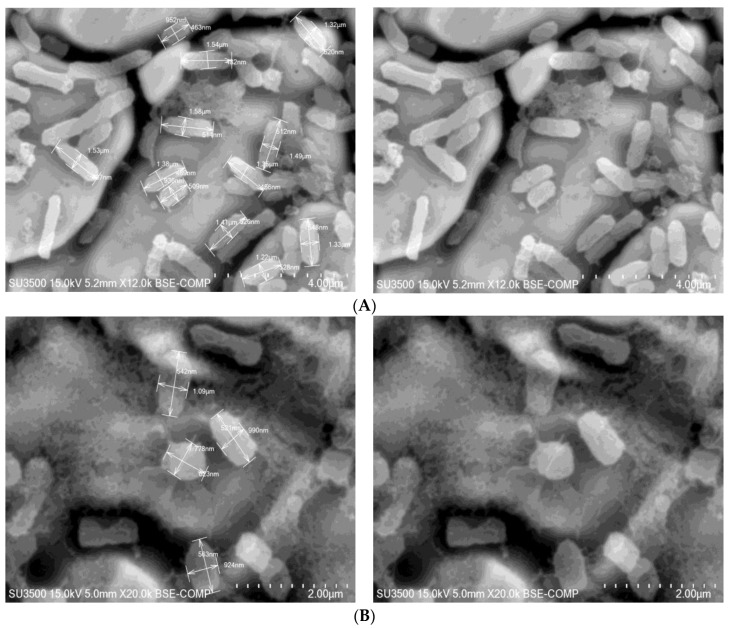
Microscopic images of *R. aquatilis* KM25 cells grown on LB medium (**A**) supplemented with 2.0 mg/mL of chlorogenic acid (**B**) (magnification ranging from ×12,000 to ×20,000).

In this work, the morphological changes in *R. aquatilis* KM25 cells were observed by SEM to highlight the antibacterial activity of chlorogenic acid. As shown in Figure 3, the exposure of *R. aquatilis* KM25 to chlorogenic acid altered its morphology. Changes in cell dimensions, mostly in length parameters from 0.778 µm to 1.09 µm, were found. In control culture, the cells displayed typical bacilliform morphology with a smooth and intact surface. The length of untreated *R. aquatilis* KM25 cells ranged from 0.952 µm to 1.53 µm (see Figure 3). Similar SEM observations were already made in *Alicyclobacillus acidoterrestris* treated with synthetic phenolic compounds and catechin/epicatechin-rich grape extract [31,32]. The results of Cai et al. [31] indicated that chlorogenic acid at concentrations of 4.0 mg/mL may have destructive effects on the cell surface and can alter the external structures of the target bacteria. This may lead to breakage of the cell membrane, resulting in leakage of protein, DNA, and RNA [31]. Additionally, Tian et al. [33] showed that chlorogenic acid induced morphological changes in bacteria; these changes in foodborne *Yersinia enterocolitica* and *Enterobacter sakazakii* were observed in a concentration-dependent manner. In the work of Tian et al. [33], a concentration of 2.5 mg/mL of chlorogenic acid caused a transition from rod-shaped cells to amorphous cells. *Y. enterocolitica* and *E. sakazakii* treated with 5.0 mg/mL chlorogenic acid were seriously damaged, and the inherent morphology of cells was lost, with a large number of contents being leaked [33].

### 3.3. Effect of Chlorogenic Acid on ftsZ, ftsA, ftsN, tolB, and M4 Gene Expression

Bacterial cell division has emerged as a new antimicrobial target pathway to counteract preservative-resistant strains [34]. Of particular interest to food microbiologists are agents that directly interact with the major cell division proteins: FtsZ, FtsA, and FtsN, thereby perturbing the function and dynamics of the cell division machinery. FtsZ acts as a coordinator of divisome formation and cytokinesis as it assembles into protofilaments to form a ring-like structure, the FtsZ-ring, at the prospective division site, where it functions as a scaffold for further members of the divisome [34]. FtsZ polymerization is dynamic and regulated by nucleotide guanosine-5′-triphosphate (GTP) hydrolysis [35]. As the cell cycle proceeds, the divisome constricts and synthesizes septal peptidoglycan to allow for septum formation and cytokinesis [34]. FtsA is a member of the actin/Hsp70/sugar kinase ATPase superfamily; it shares a universal structure containing 1A, 1C, 2A, and 2B subdomains [36]. In well-recognized *Escherichia coli*, the 1C domain of FtsA is necessary for interaction, polymerization, membrane association, and late divisome protein recruitment [37]. The 2B subdomain interacts with the cell division protein FtsZ [38]. FtsN, the last protein recruited to the divisome, initiates FtsZ-ring constriction [36].

In this work, RT–qPCR experiments were carried out to gain insight into the mechanism of growth inhibition in *R. aquatilis* KM25. Flow cytometry experiments showed that exposure in *R. aquatilis* KM25 to chlorogenic acid at a concentration of 2.0 mg/mL promoted the predominance of the subpopulation of injured cells. To clarify whether the mechanism involved in proliferation was disrupted in cells, RT–qPCR assays were carried out. *R. aquatilis* KM25 cultured in modified LB medium served as a reference. Transcriptional changes in the examined genes were calculated as the relative quantity normalized to the expression of the 16S gene.

Chlorogenic acid caused a significant reduction in the mRNA levels of the *ftsZ*, *ftsA,* and *ftsN* genes (−2.5, −1.5 and −2.0, respectively) (Table 2). Our results are in agreement with the observations of Herman et al. [39], who showed that *ftsZ* transcription was inhibited by cinnamon oil, which disrupted the *Staphylococcus aureus* division process at the level of septum synthesis. Chlorogenic acid is the most abundant phenolic compound in cinnamon oil [40] and demonstrates general antimicrobial activity against *S. aureus* strains [41]. Additionally, phytochemicals can reduce FtsZ polymerization and inhibit its GTPase activity. These processes are crucial for the formation of the Z-ring, and abnormalities in any of these functions could lead to cell death [42].

We further verified the effect of 2.0 mg/mL chlorogenic acid on the mRNA level of the *tolB* gene. TolB is a periplasmic component of the Tol-Pal system, a multiprotein complex present in almost all Gram-negative bacteria that connects the cytoplasmic (or inner) membrane with the outer membrane [43]. The system plays a relevant role in the maintenance of cell envelope integrity and in the cell division process [44]. Genes of the Tol-Pal system are also involved in the phenotypes of bacilli [45].

To the best of our knowledge, this is the first study evaluating the effect of chlorogenic acid on *tolB* gene expression. In this work, the examined concentration of chlorogenic acid downregulated the expression of the *tolB* gene (−1.5) of *R. aquatilis* KM25. This result agrees with the study of Li et al. [46], revealing that deletion of *tolB* influences cell morphology; in LB medium, the Δ*tolB* mutant of *Salmonella choleraesuis* grew in chains (3 to 10 cells) of coccobacilli. In contrast, under the same conditions, wild-type *S. choleraesuits* C78-3 grew as single rods. Additionally, the Δ*tolB1* mutant of *Erwinia chrysanthemi* formed short bacilli; the size of those cells and the position of the septa were highly irregular [47]. Moreover, Δ*tolB* mutants exhibited increased susceptibility to antimicrobials [46] and were unable to cause fatal infections [47]. These results highlight that natural agents are a promising antimicrobial food additive to improve product quality and safety [48].

The study also verified the effect of chlorogenic acid on the expression of the *M4* gene, which encodes a zinc-dependent metalloproteinase that degrades proteins and peptides for bacterial nutrition [49]. Proteolysis of fish muscles occurs even during refrigerated storage, so additional solutions are needed to prevent bacterial spoilage. In this work, the examined concentration of chlorogenic acid downregulated the expression of the *M4* gene (−1.5) in *R. aquatilis* KM25 at all time points tested. Wang et al. [24] showed that chlorogenic acid, through its effects on the quorum sensing system, inhibits protease production in *Pseudomonas aeruginosa* PA01. Computational modeling revealed that chlorogenic acid can form hydrogen bonds with quorum sensing receptors LasR, RhlR, and PqsR, and this process leads to a wide effect on transcriptional regulation of the bacterial quorum sensing system itself and the quorum sensing controlled genes related to it. Due to this metabolic disruption, an antibacterial effect can be achieved, and sensory changes in seafood can be delayed [24].

### 3.4. Effect of Chlorogenic Acid on R. aquatilis KM25 Growth in Salmon-Based Samples

In the final stage of this work, the antimicrobial effect of chlorogenic acid against the food-associated *R. aquatilis* KM25 strain was verified in in situ experiments. These results were also compared to those from the synthetic preservative benzoic acid on *R. aquatilis* KM25 growth. The results summarized in Table 3 directly demonstrate the inhibitory effect of chlorogenic acid on *R. aquatilis* KM25 proliferation. The agent inhibited between 85% and 90% of bacterial growth, and this effect was independent of storage time. A similar effect was noted in samples treated with benzoic acid; *R. aquatilis* KM25 growth inhibition was 85–95% (Table 3). These results are consistent with the work of Tian et al. [50], who also reported the inhibitory effects of chlorogenic acid (2.5 mg/mL) on *Bacillus cereus* and *Micrococcus luteus* counts in skim milk and pork samples. Phenolic acids affect both the physiology and gene expression of microorganisms; they inhibit the synthesis of key proteins involved in bacterial survival in stored products [51]. Chlorogenic acid also showed activity in vitro against *E. coli* O157:H7, *Proteus vulgaris*, and *Pseudomonas aeruginosa*, with minimum inhibitory concentrations (MICs) ranging from 0.008 to 10 mg/mL [52,53]. Furthermore, the antimicrobial activity of chlorogenic acid has been tested in probiotic bacteria; the agent did not inhibit *Bifidobacterium lactis*, *Lactobacillus crispatus*, *Lactobacillus johnsonii*, *Lactobacillus paracasei*, *Lactobacillus plantarum*, *Lactobacillus reuteri*, or *Lactobacillus rhamnosus* at concentrations up to 10 mg/mL. The resistance of these probiotics encourages the potential use of chlorogenic acid as a biopreservative in value-added food products [54].

### 3.5. Effect of Chlorogenic Acid on the Biochemical Parameters of Salmon-Based Samples

Reducing microbial growth benefits product quality and reduces the formation of TVB-N/TMA-N during storage of fish-based samples [55]. In the current study, biochemical determinations of salmon-based samples allowed verification of the preservative properties of chlorogenic acid.

TVB-N is a quality indicator of freshness, which is a measurement of basic volatile compounds recovered by distilling fish muscle under alkaline conditions [56]. The concentration of TVB-N in freshly caught fish is typically between 10 and 20 mg/100 g, whereas depending on the fish species, values of 25–35 mg/100 g in fish are considered the acceptable limit for stored fish [57].

The TVB-N content of salmon-based samples is depicted in Table 4. The TVB-N values of the control salmon-based samples (40 mg/100 g) exceeded the upper level of the maximum permissible limit of acceptability in 72 h of storage; in 120 h of storage, the TVB-N parameter was 45 mg/100 g, respectively. Supplementation of the marinate with selected concentrations of chlorogenic acid significantly reduced the formation of TVB-N content throughout the storage time; in these samples, the level of the TVB-N parameter averaged between 10 and 28 mg/100 g (Table 4). Similar values of the TVB-N parameter (7.5–25.0 mg/100 g) were also recorded in samples with benzoic acid-supplemented marinades (Table 4). These results highlight that a reduction in microbial *R. aquatilis* KM25 growth significantly reduced TVB-N formation during storage, extending the shelf life of model products. Similarly, Ozyurt et al. [58] revealed that the phenolics of rosemary extracts limited TVB-N formation in sardine muscles. Additionally, Arulkumar et al. [56] detected reduced TVB-N levels in Indian mackerel during storage due to the inclusion of the phenolic-rich red alga *Gracilaria verrucosa* in the products. Application of betel leaf (*Piper betle*) extracts on sardine muscle reduced *Enterobacteriaceae, Pseudomonas* spp., and *Aeromonas* spp. growth and limited TVB-N formation [56].

Finally, in this work, the TMA-N values were used as quality control parameters for salmon-based products. The initial levels of TMA-N in the control sample at 1 h and 24 h of storage were 2.5 mg/100 g and 5.0 mg/100 g, respectively (Table 4). The progressive formation of TMA-N was observed at subsequent storage points and was 28.5 mg/100 g (at 48 h of storage), 39.0 mg/100 g (at 72 h of storage), and 45.0 mg/100 g (at 120 h of storage). The presence of chlorogenic acid in salmon marinate led to remarkable inhibition of TMA-N formation in model products; TMA-N values were in the range of 20.5 mg/100 g–20.0 mg/100 g at 72 h and 120 h of storage, respectively. Similarly, inhibition of TMA-N formation was found in samples with the addition of benzoic acid; these values were in the range of 15.5 mg/100 g–20.0 mg/100 g at 72 h and 120 h of storage (Table 4). In agreement with the current study, previous reports by Ozyurt et al. [58] and Viji et al. [59] showed that the inhibition of TMA-N formation could be enhanced by incorporating bioactive agents in ice during chilled storage of marine fish; these studies used rosemary extracts during chilled storage of sardines [60] and mint extract applied to Indian mackerel [59].

In conclusion, chlorogenic acid at 2.0 mg/mL exhibits antimicrobial potential by affecting the expression of genes involved in the *R. aquatilis* KM25 division process and proteolytic activity. SEM observations also confirmed the effect of the examined agent on morphological changes in *R. aquatilis* KM25. The supplementation of fish marinate with chlorogenic acid reduced bacterial growth in model salmon-based products and significantly delayed the development of biochemical markers of fish deterioration (TVB-N and TMA-N). These results were consistent with the effect observed when benzoic acid was introduced into the model product. Our findings reveal the potential of chlorogenic acid as an alternative food preservative; it can increase the safety, shelf life, and quality of fish and other food products. The possibility of using chlorogenic acid in food production also aligns with the current trend of abandoning and/or reducing the use of synthetic preservatives.

## Figures and Tables

**Table 1:** Primers used in RT–qPCR experiments.

Primer	Sequence (5′→3′)	Amplified Region
16S_F	GGAGACTGCCGGTGACAAAC	16S rRNA gene (universal primers)
16S_R	TGTAGCCCAGGCCGTAAGG	
FTSZ_F	GTAGGCCAGACGATTCAG	*ftsZ* gene
FTSZ_R	GGGCTTCACGATCTTCTT	
FTSA_F	CAAGTCGACGGACAGAAAA	*ftsA* gene
FTSA_R	CTGCCCACGCCAATAATA	
FTSN_F	GTACTGGTGGTGTTTGTC	*ftsN* gene
FTSN_R	TTCTTCTGGCTTAGGTGG	
TOLB_F	GTTCGCATTGAAATTACCC	*tolB* gene
TOLB_R	TACCACCCACATCTTCAG	
M4_F	TCCCGCCCTACATGCTTA	*M4* gene
M4_R	CTTTGGCTGTCACGATCTT	

**Table 2:** RT–qPCR confirmation of relative expression level of proliferation-related genes of *Rahnella aquatilis* KM25 grown in modified LB medium with 2.0 mg/mL of chlorogenic acid.

Time of Incubation (h)	Log_2_ (Relative Quantity)				
	*ftsZ*	*ftsA*	*ftsN*	*tolB*	*M4*
24	−2.5	−1.5	−2.0	−1.5	−1.5
72	−2.5	−1.0	−2.5	−1.5	−1.5

**Table 3:** Growth inhibition (%) of *Rahnella aquatilis* KM25 in model salmon fillets during refrigerated storage. Additionally, shown is the significance of the experimental data determined by ANOVA (*p*, *F*). Means with the same letter do not differ significantly.

Storage Time (h)	Growth Inhibition (%)	
	Salmon Fillets with Chlorogenic Acid (*p* = 0.105; *F* = 2.972)	Salmon Fillets with Benzoic Acid (*p* = 0.001; *F* = 8.163)
1	85 ^a^ ± 1.5	95 ^a^ ± 1.0
24	85 ^a^ ± 1.5	95 ^a^ ± 2.0
48	90 ^a^ ± 2.0	95 ^a^ ± 1.0
72	90 ^a^ ± 1.5	95 ^a^ ± 1.5
120	80 ^a^ ± 2.5	85 ^a b^ ± 2.0

**Table 4:** Assessment of TVB-N (mg/100 g) and TMA-N (mg/100 g) of model salmon fillets during refrigerated storage. In addition, shown is the significance of the experimental data determined by ANOVA (*p*, *F*). Means with the same letter do not differ significantly.

Storage Time (h)		TVB-N		TMA-N		
	Salmon Fillets with Chlorogenic Acid (*p* = 0.0041; *F* = 8.225)	Salmon Fillets with Benzoic Acid (*p* < 0.001; *F* = 17.25)	Control (*p* = 0.0223; *F* = 5.725)	Salmon Fillets with Chlorogenic Acid (*p* < 0.0001; *F* = 33.52)	Salmon Fillets with Benzoic Acid (*p* < 0.001; *F* = 16.99)	Control (*p* < 0.0001; *F* = 15.39)
1	10 ^a^ ± 1.5	7.5 ^a^ ± 3.5	30 ^a^ ± 1.0	2.5 ^a^ ± 1.0	2.0 ^a^ ± 1.0	2.5 ^a^ ± 1.0
24	20 ^a^ ± 3.5	18.0 ^a^ ± 4.5	37 ^a^ ± 2.0	3.0 ^a^ ± 1.5	2.0 ^a^ ± 1.0	5.0 ^a^ ± 1.0
48	22 ^b,d^ ± 2.0	18.5 ^b,d^ ± 1.5	38 ^b,d^ ± 1.5	16.5 ^b,d^ ± 2.0	10.0 ^b,d^ ± 3.0	28.5 ^b,d^ ± 5.0
72	25 ^c,d^ ± 1.5	22 ^c,d^ ± 2.5	40 ^c,d^ ± 2.5	20.5 ^c,e^ ± 2.0	15.5 ^c,e^ ± 2.0	39.0 ^c,d^ ± 5.5
120	28 ^c,e^ ± 0.5	25.5 ^c,e^ ± 2.5	45 ^d,e^ ± 3.5	25.0 ^c,e^ ± 3.5	20.0 ^c,e^ ± 2.0	45.0 ^c,d^ ± 6.5

## Data Availability

The data presented in this study are available on request from the corresponding author.
